# Isolation and crystal structure of lawinal

**DOI:** 10.1107/S2056989020016540

**Published:** 2021-01-01

**Authors:** Virayu Suthiphasilp, Pornphimol Meesakul, Christopher Richardson, Stephen G. Pyne, Surat Laphookhieo

**Affiliations:** a Center of Chemical Innovation for Sustainability (CIS) and School of Science, Mae Fah Luang University, Chiang Rai 57100, Thailand; b School of Chemistry and Molecular Bioscience, University of Wollongong, Wollongong, New South Wales 2522, Australia

**Keywords:** crystal structure, natural product, lawinal, flavanone

## Abstract

The structure of the natural product lawinal [systematic name: (−)-(2*S*)-5,7-dihy­droxy-6-methyl-4-oxo-2-phenyl­chromane-8-carbaldehyde, C_17_H_14_O_5_] at 150 K is reported. The compound crystallizes with monoclinic (*I*2) symmetry and with *Z*′ = 2. The absolute configuration could not be determined reliably from X-ray analysis only. However, our analysis returns the *S*-configuration at the C-2 position, consistent with previous stereochemical assignment from specific rotation. The independent mol­ecules form into alternating hydrogen-bonded chains with C—H⋯O=CH inter­molecular linkages that run parallel to the crystallographic *a* axis and are extended into the *ac* plane by π–π inter­actions between their phenyl substituents.

## Abstract 2

The crystal structure of the natural product lawinal is reported. The compound crystallizes with monoclinic (*I*2) symmetry and with *Z*′ = 2.

## Chemical context   

The small flowering plants of the *Desmos* genus belong to the Annona­ceae family, which comprises about 33 species and is distributed widely throughout Southern Asia and northern Australia (Brophy *et al.*, 2002 ▸; Clement *et al.*, 2017 ▸). Several species of this genus have been used as Chinese folk medicines (Wu *et al.*, 2003 ▸). The aerial part of *D. chinensis* has been used as an analgesic agent, and to treat vertigo, and parturition (Kummee & Intaraksa, 2008 ▸; Rahman *et al.*, 2003 ▸). In Thailand it is widely used traditionally to treat fever and dysentery (Bunyapraphatsara *et al.*, 2000 ▸). The petroleum ether extracts of *D. cochinchinensis* roots have mainly been explored for their anti­malarial activity (Liao *et al.*, 1989 ▸). The *Desmos* genus is well known as an abundant source of flavonoids (Meesakul *et al.*, 2019 ▸; Bajgai *et al.*, 2011 ▸; Kuo *et al.*, 2015 ▸), and their 2*S* absolute configuration has been commonly found (Meesakul *et al.*, 2019 ▸; Kuo *et al.*, 2015 ▸). Flavonoids exhibit inter­esting biological activities, including inhibition of HIV-1 replication in H9 lymphocytic cells (Wu *et al.*, 2003 ▸), anti­bacterial properties (Liao *et al.*, 1989 ▸) and show activities as α-glucosidase inhibitors (Meesakul *et al.*, 2019 ▸), anti­oxidants (Miller, 1996 ▸), aromatase and lipoxygenase inhibitors (Bajgai *et al.*, 2011 ▸).
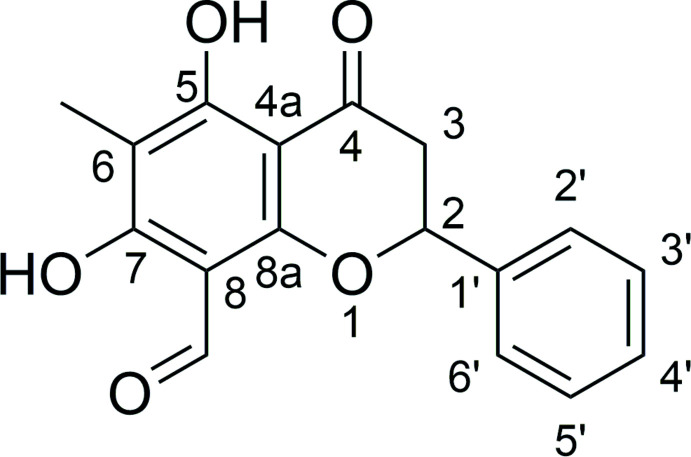


Herein, we report the isolation and crystal structure of the flavonoid, (−)(2*S*)-5,7-dihy­droxy-6-methyl-4-oxo-2-phenyl­chromane-8-carbaldehyde, commonly known as lawinal, isolated from the twig extract of *D. dumosus*.

## Structural commentary   

**Figure 1:**
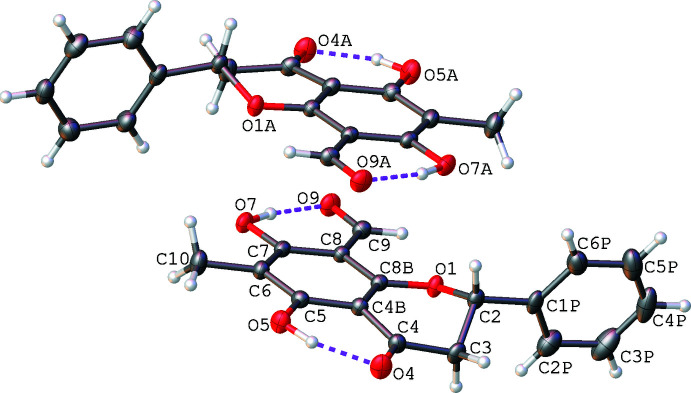
The contents of the asymmetric unit with complete atom labelling of one mol­ecule and selected heteroatom labelling of the second mol­ecule, for clarity. Intra­molecular hydrogen bonds are shown as dashed magenta lines. Displacement ellipsoids are plotted at the 50% probability level.

Lawinal crystallizes in the space group *I*2 with *Z*′ = 2. Because of the large standard deviation of the Flack parameter [−0.1 (5)], the absolute configuration cannot be assigned from the X-ray data (Parsons *et al.*, 2013 ▸). We explored applying the Bayesian statistical approach promoted by Hooft *et al.* (2008 ▸). Given that the compound comes from a natural product source and thus should be enanti­opure, the analysis, as implemented in *PLATON* (Spek, 2020 ▸), returned a *P*2(true) value of 0.992 for the *S*-configuration at C2 in each mol­ecule (Fig. 1 ▸). This is consistent with the stereochemical assignment by the method of specific rotation (Prawat *et al.*, 2012 ▸; Wu *et al.*, 2005 ▸).

**Figure 2:**
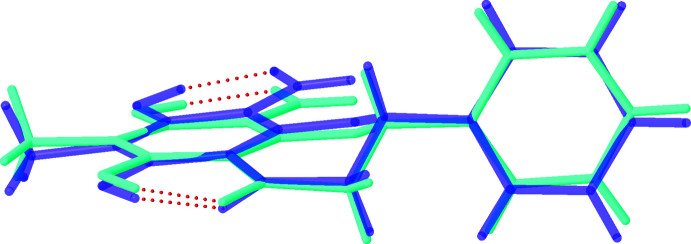
An overlay of the independent mol­ecules in the asymmetric unit. The dotted lines represent the intra­molecular hydrogen bonds.

The unique mol­ecules adopt extremely similar conformations and an overlay of the mol­ecular structures is shown in Fig. 2 ▸. The hydroxyl groups attached to C5 and C7 on each unique mol­ecule act as hydrogen-bond donors to the ketone and aldehyde functionalities, respectively. The positions of the hydroxyl hydrogen atoms were refined, the relatively long *D*—H distances (Table 1 ▸) indicating strong intra­molecular stabil­ization. The hydrogen bond O7—H7⋯O9 is responsible for bringing the aldehyde group into approximate coplanarity with the chromanone ring system. In contrast, the phenyl substituents attached to C2 in each mol­ecule are approximately orthogonal to the chromanone ring systems [plane-to-plane angles of 99.4 (1) and 97.5 (1)° to the phenyl rings of the chromanones].

## Supra­molecular features   

**Figure 3:**
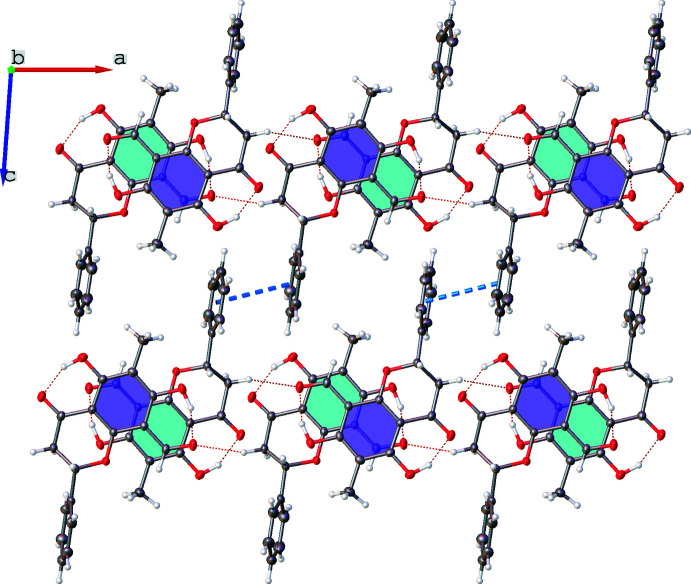
A view parallel to the crystallographic *b*-axis, with intra­molecular and inter­molecular hydrogen bonds shown as dotted red lines and the π–π inter­actions as dashed blue lines. The inter­molecular hydrogen bonds link mol­ecules into chains propagating along the crystallographic *a*-axis direction and the π–π inter­actions link the hydrogen-bonded chains into two-dimensional sheets in the crystallographic *ac* plane. Displacement ellipsoids are plotted at the 50% probability level.

The shortest inter­molecular contacts to hydrogen-bond acceptors of the unique mol­ecules come from the pseudo-equatorial C—H bonds in the CH_2_ moieties of the chromanone rings to the aldehyde oxygen atoms, O9 and O9*A* (Table 1 ▸). These C—H⋯O=CH connections assemble the unique mol­ecules into alternating chains that propagate parallel to the crystallographic *a*-axis, as shown in Fig. 3 ▸. The supra­molecular alignment of these hydrogen bonded chains are controlled by π–π inter­actions of phenyl rings from adjacent chains. This links the chains into two-dimensional sheets in the *ac* plane. The plane-to-plane angle between phenyl rings is 4.7 (1)° and the distance from plane centroid to plane centroid, as indicated by the blue dashed line in Fig. 3 ▸, is 3.821 (2) Å.

## Synthesis and crystallization   

**Plant Material**

*Desmos dumosus* twigs were collected from Doi Tung, Chiang Rai Province, Thailand, in February 2016. The plant was identified by Mr Matin Van de Bult (Doi Tung Development Project, Chiang Rai, Thailand). The specimen (MFU-NPR0110) was deposited at Mae Fah Luang University’s Natural Products Research Laboratory.

**Extraction and Isolation**

Air-dried twigs of *D. dumosus* (7.00 kg) were extracted for three days at room temperature with EtOAc (20 L). Removal of the solvent under reduced pressure provided the crude extract (92.7 g), which was subjected to column chromatography over silica gel using a gradient of hexa­nes and EtOAc (100% hexa­nes to 100% EtOAc) to afford 12 fractions (D1-D12). Fraction D5 (7.70 g) was further fractionated by column chromatography over Sephadex-LH 20 resin eluting with 100% MeOH to provide nine subfractions (D5A-D5I). Subfraction D5E (1.45 g) was further separated by column chromatography over silica gel (1:4, *v*/*v* EtOAc/hexa­nes) to give lawinal (35.5 mg) as a faint yellow-coloured solid.

**Crystallization and characterization data**

Crystals grew from slow evaporation of a 1:4 di­chloro­methane:methanol solution. M.p. 488–489 K [Lit. (Prawat *et al.*, 2012 ▸) 487 K]; [α]_D_^25^ −52.4 (*c* 0.2, CH_2_Cl_2_); ECD (3.4 × 10^−4^) *λ*_max_ (Δɛ) 298 (+4.66), 276 (−4.88), and 228 (+3.82); ^1^H NMR (CDCl_3_, 500 MHz) *δ*_H_ 12.85 (1H, *s*, OH-5), 13.00 (1H, *s*, OH-7), 10.11 (1H, *s*, C*H*O), 7.45 (5H, *m*, H-2′–H-6′), 5.57 (1H, *dd*, *J* = 13.0, 3.2 Hz, H-2), 3.16 (1H, *dd*, *J* = 17.3, 13.0 Hz, Hα-3), 2.93 (1H, *dd*, *J* = 17.3, 3.2 Hz, Hβ-3), 2.02 (3H, *s*, C*H*_3_); ^13^C NMR (CDCl_3_, 125 MHz) *δ*_C_ 6.0 (*C*H_3_), 42.8 (C-3), 80.3 (C2), 101.3 (C4a), 104.1 (C8), 105.7 (C6), 126.1 (C2′, C6′), 129.1 (C3′, C4′, C5′), 137.6 (C1′), 164.7 (C8a), 166.6 (C5), 168.8 (C7), 191.3 (*C*HO), 195.3 (C4).

## Refinement   

The data were collected using Mo *K*α radiation, therefore anomalous dispersion effects are small. The crystal structure itself is pseudo-centrosymmetric. Indeed, a structural solution can be successfully obtained in a centrosymmetric space group, although this results in an unsatisfactory refinement, with apparent disorder about the stereogenic center, as expected. The actual inversion symmetry is, of course, incompatible with the natural origin and optical activity of the compound. Crystal data, data collection and structure refinement details are summarized in Table 2 ▸. Tertiary C(H), secondary C(H,H), primary C(H,H,H) and aromatic H atoms were placed in geometrically idealized positions (C—H = 1.00, 0.99, 0.98, and 0.95 Å, respectively) and refined in riding models with *U*_iso_(H) = 1.2*U*_eq_(C) or 1.5*U*_eq_(C). The methyl group attached to C-6 was refined as a rotating body. The hy­droxy­lic H atoms were refined unconstrained in isotropic approximation.

## Supplementary Material

Crystal structure: contains datablock(s) I. DOI: 10.1107/S2056989020016540/zv2003sup1.cif

Structure factors: contains datablock(s) I. DOI: 10.1107/S2056989020016540/zv2003Isup2.hkl

PLATON Output. DOI: 10.1107/S2056989020016540/zv2003sup3.txt

Supporting information file. DOI: 10.1107/S2056989020016540/zv2003Isup4.cml

CCDC reference: 2051848

Additional supporting information:  crystallographic information; 3D view; checkCIF report

## Figures and Tables

**Table 1:** Hydrogen-bond geometry (Å, °)

*D*—H⋯*A*	*D*—H	H⋯*A*	*D*⋯*A*	*D*—H⋯*A*
C3—H3*B*⋯O9*A* ^i^	0.99	2.32	3.252 (3)	158
C3*A*—H3*AB*⋯O9^ii^	0.99	2.31	3.248 (3)	157
O7*A*—H7*A*⋯O9*A*	0.94 (3)	1.67 (3)	2.572 (2)	159 (4)
O5—H5⋯O4	0.94 (3)	1.70 (3)	2.579 (2)	154 (3)
O7—H7⋯O9	0.95 (3)	1.67 (3)	2.585 (2)	162 (3)
O5*A*—H5*A*⋯O4*A*	0.94 (3)	1.70 (3)	2.574 (2)	153 (3)

Symmetry codes: (i) 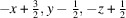; (ii) 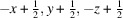.

**Table 2:** Experimental details

Crystal data	
Chemical formula	C_17_H_14_O_5_
*M*_r_	298.28
Crystal system, space group	Monoclinic, *I*2
Temperature (K)	150
*a*, *b*, *c* (Å)	18.9581 (14), 6.6461 (4), 22.4043 (16)
β (°)	94.163 (7)
*V* (Å^3^)	2815.4 (3)
*Z*	8
Radiation type	Mo *K*α
μ (mm^−1^)	0.10
Crystal size (mm)	0.42 × 0.19 × 0.16

Data collection	
Diffractometer	Rigaku XtaLAB Mini II
Absorption correction	Multi-scan (*CrysAlis PRO*; Rigaku OD, 2018 ▸)
*T*_min_, *T*_max_	0.728, 1.000
No. of measured, independent and observed [*I* > 2σ(*I*)] reflections	33601, 7914, 6451
*R*_int_	0.072
(sin θ/λ)_max_ (Å^−1^)	0.718

Refinement	
*R*[*F* ^2^ > 2σ(*F* ^2^)], *wR*(*F* ^2^), *S*	0.053, 0.144, 1.02
No. of reflections	7914
No. of parameters	415
No. of restraints	1
H-atom treatment	H atoms treated by a mixture of independent and constrained refinement
Δρ_max_, Δρ_min_ (e Å^−3^)	0.39, −0.32
Absolute structure	Flack *x* determined using 2350 quotients [(*I* ^+^)−(*I* ^−^)]/[(*I* ^+^)+(*I* ^−^)] (Parsons *et al.*, 2013 ▸)
Absolute structure parameter	−0.1 (5)

Computer programs: *CrysAlis PRO* (Rigaku OD, 2018 ▸), *SHELXS* (Sheldrick, 2008 ▸), *SHELXL* (Sheldrick, 2015 ▸) and *OLEX2* (Dolomanov *et al.*, 2009 ▸).

## supplementary crystallographic information

### Crystal data

**Table:**

C_17_H_14_O_5_	*F*(000) = 1248
*M**_r_* = 298.28	*D*_x_ = 1.407 Mg m^−^^3^
Monoclinic, *I*2	Mo *K*α radiation, λ = 0.71073 Å
*a* = 18.9581 (14) Å	Cell parameters from 12756 reflections
*b* = 6.6461 (4) Å	θ = 2.1–30.6°
*c* = 22.4043 (16) Å	µ = 0.10 mm^−^^1^
β = 94.163 (7)°	*T* = 150 K
*V* = 2815.4 (3) Å^3^	Block, clear colourless
*Z* = 8	0.42 × 0.19 × 0.16 mm

### Data collection

**Table:**

Rigaku XtaLAB Mini II diffractometer	7914 independent reflections
Radiation source: fine-focus sealed X-ray tube, Rigaku (Mo) X-ray Source	6451 reflections with *I* > 2σ(*I*)
Graphite monochromator	*R*_int_ = 0.072
ω scans	θ_max_ = 30.7°, θ_min_ = 2.2°
Absorption correction: multi-scan (CrysAlisPro; Rigaku OD, 2018)	*h* = −26→26
*T*_min_ = 0.728, *T*_max_ = 1.000	*k* = −9→9
33601 measured reflections	*l* = −32→32

### Refinement

**Table:**

Refinement on *F*^2^	Hydrogen site location: mixed
Least-squares matrix: full	H atoms treated by a mixture of independent and constrained refinement
*R*[*F*^2^ > 2σ(*F*^2^)] = 0.053	*w* = 1/[σ^2^(*F*_o_^2^) + (0.0925*P*)^2^] where *P* = (*F*_o_^2^ + 2*F*_c_^2^)/3
*wR*(*F*^2^) = 0.144	(Δ/σ)_max_ < 0.001
*S* = 1.02	Δρ_max_ = 0.39 e Å^−^^3^
7914 reflections	Δρ_min_ = −0.32 e Å^−^^3^
415 parameters	Absolute structure: Flack *x* determined using 2350 quotients [(*I*^+^)-(*I*^-^)]/[(*I*^+^)+(*I*^-^)] (Parsons *et al.*, 2013)
1 restraint	Absolute structure parameter: −0.1 (5)
Primary atom site location: structure-invariant direct methods	

### Special details

**Table:**

Geometry. All esds (except the esd in the dihedral angle between two l.s. planes) are estimated using the full covariance matrix. The cell esds are taken into account individually in the estimation of esds in distances, angles and torsion angles; correlations between esds in cell parameters are only used when they are defined by crystal symmetry. An approximate (isotropic) treatment of cell esds is used for estimating esds involving l.s. planes.

### Fractional atomic coordinates and isotropic or equivalent isotropic displacement parameters (Å^2^)

**Table:**

	*x*	*y*	*z*	*U*_iso_*/*U*_eq_	
O1	0.57571 (8)	0.2436 (3)	0.16037 (6)	0.0205 (4)	
O4	0.73983 (8)	0.1600 (3)	0.28806 (7)	0.0284 (4)	
O5	0.64990 (9)	0.1903 (3)	0.36865 (6)	0.0248 (4)	
O7	0.40722 (8)	0.2205 (3)	0.30729 (7)	0.0220 (4)	
O9	0.37112 (8)	0.2498 (3)	0.19412 (7)	0.0262 (4)	
C2	0.64822 (11)	0.3054 (4)	0.14926 (8)	0.0204 (4)	
H2	0.655815	0.445414	0.164727	0.024*	
C3	0.70093 (11)	0.1669 (4)	0.18367 (9)	0.0225 (5)	
H3A	0.693728	0.026481	0.169839	0.027*	
H3B	0.749831	0.207369	0.176389	0.027*	
C4	0.69015 (11)	0.1811 (4)	0.24939 (9)	0.0205 (4)	
C5	0.59854 (11)	0.2066 (3)	0.32465 (9)	0.0184 (4)	
C6	0.52807 (11)	0.2131 (4)	0.34014 (9)	0.0193 (5)	
C7	0.47547 (11)	0.2219 (3)	0.29309 (9)	0.0173 (4)	
C8	0.49093 (11)	0.2327 (3)	0.23198 (9)	0.0163 (4)	
C9	0.43514 (11)	0.2491 (4)	0.18501 (9)	0.0203 (5)	
H9	0.447893	0.259941	0.144908	0.024*	
C10	0.50961 (12)	0.2053 (5)	0.40436 (9)	0.0280 (6)	
H10A	0.488694	0.333896	0.415220	0.042*	
H10B	0.552530	0.180389	0.430374	0.042*	
H10C	0.475581	0.096640	0.409285	0.042*	
C4B	0.61711 (12)	0.2130 (4)	0.26415 (9)	0.0177 (4)	
C8B	0.56271 (11)	0.2306 (3)	0.21864 (9)	0.0165 (4)	
C1P	0.65328 (11)	0.3085 (4)	0.08262 (9)	0.0243 (5)	
C2P	0.64364 (13)	0.1363 (5)	0.04797 (11)	0.0366 (6)	
H2P	0.633195	0.011407	0.065971	0.044*	
C3P	0.64952 (15)	0.1489 (6)	−0.01417 (12)	0.0462 (8)	
H3P	0.642758	0.032115	−0.038366	0.055*	
C4P	0.66512 (13)	0.3311 (7)	−0.04014 (10)	0.0448 (8)	
H4P	0.669125	0.338660	−0.082103	0.054*	
C5P	0.67485 (13)	0.5008 (6)	−0.00559 (10)	0.0430 (7)	
H5P	0.685672	0.625104	−0.023760	0.052*	
C6P	0.66898 (12)	0.4918 (5)	0.05540 (10)	0.0324 (6)	
H6P	0.675579	0.609940	0.079017	0.039*	
O1A	0.42391 (8)	0.7039 (3)	0.34098 (6)	0.0201 (3)	
O4A	0.26002 (9)	0.6903 (3)	0.21057 (7)	0.0290 (4)	
O5A	0.35043 (9)	0.7514 (3)	0.13220 (7)	0.0280 (4)	
O7A	0.59290 (8)	0.7443 (3)	0.19545 (7)	0.0233 (4)	
O9A	0.62819 (8)	0.7220 (3)	0.30829 (7)	0.0267 (4)	
C2A	0.35218 (11)	0.7575 (4)	0.35631 (9)	0.0206 (5)	
H2A	0.345949	0.906404	0.352371	0.025*	
C3A	0.29726 (11)	0.6534 (4)	0.31422 (9)	0.0243 (5)	
H3AA	0.299403	0.506440	0.321265	0.029*	
H3AB	0.249480	0.700565	0.322737	0.029*	
C4A	0.30940 (12)	0.6959 (4)	0.25019 (9)	0.0211 (4)	
C5A	0.40185 (12)	0.7439 (3)	0.17646 (9)	0.0202 (5)	
C6A	0.47233 (12)	0.7487 (4)	0.16168 (9)	0.0212 (5)	
C7A	0.52461 (11)	0.7395 (3)	0.20899 (9)	0.0188 (4)	
C8A	0.50869 (11)	0.7268 (3)	0.26992 (9)	0.0173 (4)	
C9A	0.56451 (11)	0.7198 (4)	0.31718 (9)	0.0204 (5)	
H9A	0.551698	0.713108	0.357350	0.024*	
C10A	0.49126 (13)	0.7599 (5)	0.09739 (9)	0.0307 (6)	
H10D	0.451210	0.712862	0.070936	0.046*	
H10E	0.532553	0.674776	0.092114	0.046*	
H10F	0.502307	0.899468	0.087429	0.046*	
C4AA	0.38250 (12)	0.7287 (4)	0.23673 (9)	0.0187 (4)	
C8AA	0.43682 (11)	0.7208 (3)	0.28291 (9)	0.0167 (4)	
C1PA	0.34755 (12)	0.6990 (4)	0.42113 (9)	0.0223 (5)	
C2PA	0.33826 (12)	0.5001 (4)	0.43833 (9)	0.0278 (5)	
H2PA	0.335466	0.396820	0.408958	0.033*	
C3PA	0.33302 (13)	0.4515 (4)	0.49835 (10)	0.0316 (6)	
H3PA	0.326462	0.315656	0.509901	0.038*	
C4PA	0.33746 (12)	0.6031 (5)	0.54122 (10)	0.0302 (6)	
H4PA	0.333388	0.570378	0.582103	0.036*	
C5PA	0.34774 (13)	0.8014 (5)	0.52490 (10)	0.0317 (6)	
H5PA	0.351439	0.904073	0.554461	0.038*	
C6PA	0.35263 (12)	0.8490 (4)	0.46478 (9)	0.0281 (5)	
H6PA	0.359485	0.984872	0.453403	0.034*	
H7A	0.616 (2)	0.742 (6)	0.2338 (15)	0.060 (11)*	
H5	0.6917 (17)	0.176 (6)	0.3492 (13)	0.049 (9)*	
H7	0.3847 (18)	0.226 (6)	0.2681 (13)	0.042 (9)*	
H5A	0.3078 (17)	0.733 (5)	0.1507 (13)	0.041 (9)*	

### Atomic displacement parameters (Å^2^)

**Table:**

	*U*^11^	*U*^22^	*U*^33^	*U*^12^	*U*^13^	*U*^23^
O1	0.0133 (7)	0.0324 (10)	0.0158 (6)	−0.0003 (6)	0.0016 (5)	−0.0005 (6)
O4	0.0144 (7)	0.0437 (11)	0.0265 (8)	0.0028 (7)	−0.0028 (6)	0.0037 (7)
O5	0.0194 (8)	0.0352 (10)	0.0193 (7)	0.0010 (7)	−0.0026 (6)	0.0004 (7)
O7	0.0152 (8)	0.0282 (10)	0.0233 (7)	−0.0001 (6)	0.0062 (6)	0.0003 (6)
O9	0.0131 (8)	0.0363 (11)	0.0295 (8)	0.0006 (7)	0.0032 (6)	0.0018 (7)
C2	0.0136 (9)	0.0281 (12)	0.0198 (9)	0.0000 (8)	0.0031 (7)	0.0003 (8)
C3	0.0126 (9)	0.0326 (13)	0.0225 (9)	0.0021 (9)	0.0028 (7)	−0.0008 (9)
C4	0.0167 (10)	0.0247 (11)	0.0200 (9)	0.0009 (8)	0.0011 (7)	0.0024 (8)
C5	0.0182 (11)	0.0191 (11)	0.0178 (9)	0.0010 (8)	−0.0002 (8)	0.0002 (8)
C6	0.0183 (11)	0.0224 (12)	0.0176 (9)	0.0003 (8)	0.0030 (8)	0.0001 (8)
C7	0.0158 (11)	0.0167 (11)	0.0199 (9)	0.0000 (8)	0.0041 (8)	−0.0001 (8)
C8	0.0139 (10)	0.0177 (11)	0.0172 (8)	0.0003 (8)	0.0017 (7)	−0.0006 (7)
C9	0.0165 (11)	0.0222 (11)	0.0221 (10)	0.0001 (8)	0.0013 (8)	−0.0009 (8)
C10	0.0227 (12)	0.0425 (16)	0.0194 (10)	−0.0009 (10)	0.0045 (9)	0.0010 (10)
C4B	0.0157 (10)	0.0203 (11)	0.0168 (9)	0.0003 (8)	−0.0005 (7)	−0.0004 (8)
C8B	0.0142 (10)	0.0170 (11)	0.0184 (9)	−0.0003 (8)	0.0014 (8)	−0.0008 (8)
C1P	0.0128 (10)	0.0411 (15)	0.0195 (9)	0.0030 (9)	0.0044 (7)	0.0003 (9)
C2P	0.0278 (13)	0.0468 (17)	0.0355 (12)	−0.0015 (12)	0.0054 (10)	−0.0102 (12)
C3P	0.0294 (14)	0.075 (2)	0.0339 (13)	0.0016 (15)	−0.0012 (10)	−0.0245 (15)
C4P	0.0223 (13)	0.090 (3)	0.0218 (10)	0.0010 (15)	0.0026 (9)	0.0013 (15)
C5P	0.0309 (14)	0.069 (2)	0.0297 (12)	0.0013 (14)	0.0033 (10)	0.0158 (14)
C6P	0.0259 (13)	0.0431 (16)	0.0286 (11)	0.0053 (11)	0.0042 (9)	0.0083 (11)
O1A	0.0137 (7)	0.0304 (9)	0.0165 (6)	0.0004 (6)	0.0017 (5)	0.0003 (6)
O4A	0.0154 (8)	0.0450 (12)	0.0261 (8)	0.0013 (7)	−0.0028 (6)	0.0000 (8)
O5A	0.0226 (9)	0.0426 (11)	0.0183 (7)	0.0018 (8)	−0.0019 (6)	−0.0020 (7)
O7A	0.0159 (8)	0.0285 (10)	0.0264 (8)	−0.0009 (6)	0.0071 (6)	−0.0023 (7)
O9A	0.0136 (8)	0.0353 (11)	0.0310 (8)	0.0012 (7)	0.0015 (6)	−0.0002 (7)
C2A	0.0155 (10)	0.0272 (12)	0.0195 (9)	0.0034 (8)	0.0035 (8)	0.0009 (8)
C3A	0.0136 (9)	0.0376 (14)	0.0218 (9)	−0.0006 (9)	0.0022 (7)	0.0000 (9)
C4A	0.0156 (10)	0.0258 (12)	0.0215 (9)	0.0022 (9)	−0.0014 (7)	−0.0023 (8)
C5A	0.0208 (11)	0.0220 (12)	0.0174 (9)	0.0010 (8)	−0.0008 (8)	−0.0022 (8)
C6A	0.0231 (12)	0.0217 (12)	0.0192 (9)	0.0004 (9)	0.0045 (8)	−0.0015 (8)
C7A	0.0150 (11)	0.0179 (11)	0.0240 (10)	−0.0005 (8)	0.0050 (8)	−0.0022 (9)
C8A	0.0153 (11)	0.0174 (11)	0.0193 (9)	0.0002 (8)	0.0015 (8)	−0.0009 (7)
C9A	0.0141 (10)	0.0230 (12)	0.0238 (10)	−0.0005 (8)	0.0003 (8)	−0.0011 (8)
C10A	0.0266 (13)	0.0455 (16)	0.0207 (10)	0.0016 (11)	0.0069 (9)	−0.0020 (10)
C4AA	0.0154 (10)	0.0230 (11)	0.0177 (9)	0.0015 (8)	−0.0001 (8)	−0.0026 (8)
C8AA	0.0151 (10)	0.0183 (11)	0.0168 (9)	0.0001 (8)	0.0015 (7)	−0.0018 (8)
C1PA	0.0156 (10)	0.0330 (13)	0.0185 (9)	0.0028 (9)	0.0030 (8)	−0.0028 (9)
C2PA	0.0306 (13)	0.0321 (13)	0.0210 (10)	0.0037 (10)	0.0030 (9)	−0.0037 (9)
C3PA	0.0332 (14)	0.0327 (14)	0.0290 (11)	0.0004 (11)	0.0030 (10)	0.0029 (10)
C4PA	0.0230 (12)	0.0446 (16)	0.0233 (10)	0.0030 (11)	0.0045 (9)	0.0020 (10)
C5PA	0.0284 (13)	0.0407 (16)	0.0259 (11)	0.0015 (11)	0.0018 (9)	−0.0113 (11)
C6PA	0.0262 (12)	0.0306 (13)	0.0278 (10)	−0.0031 (10)	0.0044 (9)	−0.0041 (10)

### Geometric parameters (Å, º)

**Table:**

O1—C2	1.473 (2)	O1A—C2A	1.470 (2)
O1—C8B	1.349 (2)	O1A—C8AA	1.346 (2)
O4—C4	1.241 (3)	O4A—C4A	1.243 (3)
O5—C5	1.339 (3)	O5A—C5A	1.340 (3)
O5—H5	0.94 (3)	O5A—H5A	0.94 (3)
O7—C7	1.355 (2)	O7A—C7A	1.351 (2)
O7—H7	0.95 (3)	O7A—H7A	0.94 (3)
O9—C9	1.245 (3)	O9A—C9A	1.238 (2)
C2—H2	1.0000	C2A—H2A	1.0000
C2—C3	1.526 (3)	C2A—C3A	1.520 (3)
C2—C1P	1.503 (3)	C2A—C1PA	1.512 (3)
C3—H3A	0.9900	C3A—H3AA	0.9900
C3—H3B	0.9900	C3A—H3AB	0.9900
C3—C4	1.504 (3)	C3A—C4A	1.496 (3)
C4—C4B	1.462 (3)	C4A—C4AA	1.456 (3)
C5—C6	1.405 (3)	C5A—C6A	1.400 (3)
C5—C4B	1.426 (3)	C5A—C4AA	1.428 (3)
C6—C7	1.399 (3)	C6A—C7A	1.399 (3)
C6—C10	1.506 (2)	C6A—C10A	1.511 (2)
C7—C8	1.423 (2)	C7A—C8A	1.422 (3)
C8—C9	1.441 (3)	C8A—C9A	1.442 (3)
C8—C8B	1.414 (3)	C8A—C8AA	1.414 (3)
C9—H9	0.9500	C9A—H9A	0.9500
C10—H10A	0.9800	C10A—H10D	0.9800
C10—H10B	0.9800	C10A—H10E	0.9800
C10—H10C	0.9800	C10A—H10F	0.9800
C4B—C8B	1.401 (3)	C4AA—C8AA	1.407 (3)
C1P—C2P	1.387 (4)	C1PA—C2PA	1.392 (4)
C1P—C6P	1.404 (4)	C1PA—C6PA	1.394 (3)
C2P—H2P	0.9500	C2PA—H2PA	0.9500
C2P—C3P	1.407 (3)	C2PA—C3PA	1.393 (3)
C3P—H3P	0.9500	C3PA—H3PA	0.9500
C3P—C4P	1.384 (5)	C3PA—C4PA	1.390 (4)
C4P—H4P	0.9500	C4PA—H4PA	0.9500
C4P—C5P	1.373 (5)	C4PA—C5PA	1.385 (4)
C5P—H5P	0.9500	C5PA—H5PA	0.9500
C5P—C6P	1.380 (3)	C5PA—C6PA	1.393 (3)
C6P—H6P	0.9500	C6PA—H6PA	0.9500
			
C8B—O1—C2	114.82 (16)	C8AA—O1A—C2A	116.23 (16)
C5—O5—H5	105.1 (18)	C5A—O5A—H5A	105.8 (18)
C7—O7—H7	99 (2)	C7A—O7A—H7A	101 (2)
O1—C2—H2	108.3	O1A—C2A—H2A	108.9
O1—C2—C3	109.37 (18)	O1A—C2A—C3A	110.40 (17)
O1—C2—C1P	107.42 (17)	O1A—C2A—C1PA	106.34 (17)
C3—C2—H2	108.3	C3A—C2A—H2A	108.9
C1P—C2—H2	108.3	C1PA—C2A—H2A	108.9
C1P—C2—C3	115.06 (18)	C1PA—C2A—C3A	113.22 (18)
C2—C3—H3A	109.9	C2A—C3A—H3AA	109.4
C2—C3—H3B	109.9	C2A—C3A—H3AB	109.4
H3A—C3—H3B	108.3	H3AA—C3A—H3AB	108.0
C4—C3—C2	109.05 (17)	C4A—C3A—C2A	111.28 (19)
C4—C3—H3A	109.9	C4A—C3A—H3AA	109.4
C4—C3—H3B	109.9	C4A—C3A—H3AB	109.4
O4—C4—C3	121.76 (19)	O4A—C4A—C3A	121.2 (2)
O4—C4—C4B	122.81 (18)	O4A—C4A—C4AA	122.38 (19)
C4B—C4—C3	115.38 (19)	C4AA—C4A—C3A	116.23 (19)
O5—C5—C6	118.33 (17)	O5A—C5A—C6A	118.67 (18)
O5—C5—C4B	119.06 (18)	O5A—C5A—C4AA	118.64 (19)
C6—C5—C4B	122.6 (2)	C6A—C5A—C4AA	122.7 (2)
C5—C6—C10	121.7 (2)	C5A—C6A—C10A	121.5 (2)
C7—C6—C5	116.99 (17)	C7A—C6A—C5A	117.11 (18)
C7—C6—C10	121.26 (19)	C7A—C6A—C10A	121.33 (19)
O7—C7—C6	117.62 (17)	O7A—C7A—C6A	117.80 (18)
O7—C7—C8	119.6 (2)	O7A—C7A—C8A	119.4 (2)
C6—C7—C8	122.82 (18)	C6A—C7A—C8A	122.82 (18)
C7—C8—C9	121.00 (18)	C7A—C8A—C9A	120.75 (18)
C8B—C8—C7	118.1 (2)	C8AA—C8A—C7A	118.3 (2)
C8B—C8—C9	120.86 (17)	C8AA—C8A—C9A	120.95 (18)
O9—C9—C8	123.58 (18)	O9A—C9A—C8A	123.61 (19)
O9—C9—H9	118.2	O9A—C9A—H9A	118.2
C8—C9—H9	118.2	C8A—C9A—H9A	118.2
C6—C10—H10A	109.5	C6A—C10A—H10D	109.5
C6—C10—H10B	109.5	C6A—C10A—H10E	109.5
C6—C10—H10C	109.5	C6A—C10A—H10F	109.5
H10A—C10—H10B	109.5	H10D—C10A—H10E	109.5
H10A—C10—H10C	109.5	H10D—C10A—H10F	109.5
H10B—C10—H10C	109.5	H10E—C10A—H10F	109.5
C5—C4B—C4	120.9 (2)	C5A—C4AA—C4A	121.3 (2)
C8B—C4B—C4	120.43 (17)	C8AA—C4AA—C4A	119.93 (18)
C8B—C4B—C5	118.34 (19)	C8AA—C4AA—C5A	118.26 (19)
O1—C8B—C8	116.75 (19)	O1A—C8AA—C8A	116.55 (19)
O1—C8B—C4B	122.21 (18)	O1A—C8AA—C4AA	122.63 (18)
C4B—C8B—C8	121.03 (18)	C4AA—C8AA—C8A	120.81 (18)
C2P—C1P—C2	122.0 (2)	C2PA—C1PA—C2A	121.8 (2)
C2P—C1P—C6P	119.7 (2)	C2PA—C1PA—C6PA	119.2 (2)
C6P—C1P—C2	118.3 (2)	C6PA—C1PA—C2A	119.0 (2)
C1P—C2P—H2P	120.4	C1PA—C2PA—H2PA	119.8
C1P—C2P—C3P	119.2 (3)	C1PA—C2PA—C3PA	120.5 (2)
C3P—C2P—H2P	120.4	C3PA—C2PA—H2PA	119.8
C2P—C3P—H3P	119.9	C2PA—C3PA—H3PA	120.2
C4P—C3P—C2P	120.2 (3)	C4PA—C3PA—C2PA	119.6 (3)
C4P—C3P—H3P	119.9	C4PA—C3PA—H3PA	120.2
C3P—C4P—H4P	119.8	C3PA—C4PA—H4PA	119.7
C5P—C4P—C3P	120.4 (2)	C5PA—C4PA—C3PA	120.6 (2)
C5P—C4P—H4P	119.8	C5PA—C4PA—H4PA	119.7
C4P—C5P—H5P	119.8	C4PA—C5PA—H5PA	120.3
C4P—C5P—C6P	120.4 (3)	C4PA—C5PA—C6PA	119.4 (2)
C6P—C5P—H5P	119.8	C6PA—C5PA—H5PA	120.3
C1P—C6P—H6P	119.9	C1PA—C6PA—H6PA	119.7
C5P—C6P—C1P	120.2 (3)	C5PA—C6PA—C1PA	120.6 (2)
C5P—C6P—H6P	119.9	C5PA—C6PA—H6PA	119.7
			
O1—C2—C3—C4	−59.6 (2)	O1A—C2A—C3A—C4A	−53.9 (3)
O1—C2—C1P—C2P	−62.0 (3)	O1A—C2A—C1PA—C2PA	−77.6 (3)
O1—C2—C1P—C6P	118.7 (2)	O1A—C2A—C1PA—C6PA	102.6 (2)
O4—C4—C4B—C5	−4.7 (4)	O4A—C4A—C4AA—C5A	−3.9 (4)
O4—C4—C4B—C8B	−178.3 (2)	O4A—C4A—C4AA—C8AA	−175.9 (2)
O5—C5—C6—C7	−177.4 (2)	O5A—C5A—C6A—C7A	−179.7 (2)
O5—C5—C6—C10	0.9 (4)	O5A—C5A—C6A—C10A	−0.6 (4)
O5—C5—C4B—C4	6.4 (3)	O5A—C5A—C4AA—C4A	7.9 (3)
O5—C5—C4B—C8B	−179.9 (2)	O5A—C5A—C4AA—C8AA	180.0 (2)
O7—C7—C8—C9	2.1 (3)	O7A—C7A—C8A—C9A	0.2 (3)
O7—C7—C8—C8B	−179.0 (2)	O7A—C7A—C8A—C8AA	−179.6 (2)
C2—O1—C8B—C8	161.36 (19)	C2A—O1A—C8AA—C8A	162.39 (19)
C2—O1—C8B—C4B	−19.5 (3)	C2A—O1A—C8AA—C4AA	−18.7 (3)
C2—C3—C4—O4	−148.6 (2)	C2A—C3A—C4A—O4A	−153.9 (2)
C2—C3—C4—C4B	33.8 (3)	C2A—C3A—C4A—C4AA	30.6 (3)
C2—C1P—C2P—C3P	−179.6 (2)	C2A—C1PA—C2PA—C3PA	−178.9 (2)
C2—C1P—C6P—C5P	179.3 (2)	C2A—C1PA—C6PA—C5PA	179.2 (2)
C3—C2—C1P—C2P	60.1 (3)	C3A—C2A—C1PA—C2PA	43.9 (3)
C3—C2—C1P—C6P	−119.3 (2)	C3A—C2A—C1PA—C6PA	−136.0 (2)
C3—C4—C4B—C5	172.9 (2)	C3A—C4A—C4AA—C5A	171.6 (2)
C3—C4—C4B—C8B	−0.7 (3)	C3A—C4A—C4AA—C8AA	−0.4 (3)
C4—C4B—C8B—O1	−8.2 (3)	C4A—C4AA—C8AA—O1A	−6.9 (3)
C4—C4B—C8B—C8	170.9 (2)	C4A—C4AA—C8AA—C8A	172.0 (2)
C5—C6—C7—O7	177.5 (2)	C5A—C6A—C7A—O7A	−179.9 (2)
C5—C6—C7—C8	−2.6 (3)	C5A—C6A—C7A—C8A	−0.3 (3)
C5—C4B—C8B—O1	178.0 (2)	C5A—C4AA—C8AA—O1A	−179.1 (2)
C5—C4B—C8B—C8	−2.9 (3)	C5A—C4AA—C8AA—C8A	−0.3 (3)
C6—C5—C4B—C4	−172.4 (2)	C6A—C5A—C4AA—C4A	−171.3 (2)
C6—C5—C4B—C8B	1.3 (4)	C6A—C5A—C4AA—C8AA	0.8 (4)
C6—C7—C8—C9	−177.7 (2)	C6A—C7A—C8A—C9A	−179.3 (2)
C6—C7—C8—C8B	1.2 (3)	C6A—C7A—C8A—C8AA	0.8 (3)
C7—C8—C9—O9	−1.9 (4)	C7A—C8A—C9A—O9A	−0.8 (4)
C7—C8—C8B—O1	−179.16 (19)	C7A—C8A—C8AA—O1A	178.5 (2)
C7—C8—C8B—C4B	1.7 (3)	C7A—C8A—C8AA—C4AA	−0.5 (3)
C9—C8—C8B—O1	−0.3 (3)	C9A—C8A—C8AA—O1A	−1.4 (3)
C9—C8—C8B—C4B	−179.5 (2)	C9A—C8A—C8AA—C4AA	179.7 (2)
C10—C6—C7—O7	−0.8 (3)	C10A—C6A—C7A—O7A	1.1 (3)
C10—C6—C7—C8	179.0 (2)	C10A—C6A—C7A—C8A	−179.4 (2)
C4B—C5—C6—C7	1.3 (3)	C4AA—C5A—C6A—C7A	−0.5 (4)
C4B—C5—C6—C10	179.7 (2)	C4AA—C5A—C6A—C10A	178.6 (2)
C8B—O1—C2—C3	53.6 (2)	C8AA—O1A—C2A—C3A	49.0 (3)
C8B—O1—C2—C1P	179.10 (19)	C8AA—O1A—C2A—C1PA	172.20 (19)
C8B—C8—C9—O9	179.3 (2)	C8AA—C8A—C9A—O9A	179.0 (2)
C1P—C2—C3—C4	179.4 (2)	C1PA—C2A—C3A—C4A	−173.03 (19)
C1P—C2P—C3P—C4P	0.3 (4)	C1PA—C2PA—C3PA—C4PA	−0.3 (4)
C2P—C1P—C6P—C5P	0.0 (4)	C2PA—C1PA—C6PA—C5PA	−0.7 (4)
C2P—C3P—C4P—C5P	−0.1 (4)	C2PA—C3PA—C4PA—C5PA	−0.7 (4)
C3P—C4P—C5P—C6P	−0.2 (4)	C3PA—C4PA—C5PA—C6PA	0.9 (4)
C4P—C5P—C6P—C1P	0.3 (4)	C4PA—C5PA—C6PA—C1PA	−0.3 (4)
C6P—C1P—C2P—C3P	−0.3 (4)	C6PA—C1PA—C2PA—C3PA	0.9 (4)

### Hydrogen-bond geometry (Å, º)

**Table:**

*D*—H···*A*	*D*—H	H···*A*	*D*···*A*	*D*—H···*A*
C3—H3*B*···O9*A*^i^	0.99	2.32	3.252 (3)	158
C3*A*—H3*AB*···O9^ii^	0.99	2.31	3.248 (3)	157
O7*A*—H7*A*···O9*A*	0.94 (3)	1.67 (3)	2.572 (2)	159 (4)
O5—H5···O4	0.94 (3)	1.70 (3)	2.579 (2)	154 (3)
O7—H7···O9	0.95 (3)	1.67 (3)	2.585 (2)	162 (3)
O5*A*—H5*A*···O4*A*	0.94 (3)	1.70 (3)	2.574 (2)	153 (3)

Symmetry codes: (i) −*x*+3/2, *y*−1/2, −*z*+1/2; (ii) −*x*+1/2, *y*+1/2, −*z*+1/2.
